# Association of Some High-Risk Mucosal Types of *Human Papillomavirus* with Cutaneous Squamous Cell Carcinoma in an Iranian Population

**DOI:** 10.30699/ijp.2019.101544.2011

**Published:** 2019-09-22

**Authors:** Hassan Ehteram, Mohaddeseh Sadat Mousavian, Tahereh Mazoochi, Tahereh Khamehchian, Mohammad Karimian

**Affiliations:** 1 *Department of Pathology, Faculty of Medicine, Kashan University of Medical Sciences, Kashan, Iran*; 2 *Gametogenesis Research Center, Kashan University of Medical Sciences, Kashan, Iran *; 3 *Anatomical Sciences Research Center, Kashan University of Medical Sciences, Kashan, Iran*

**Keywords:** Squamous cell carcinoma, Human papillomavirus, Association study, Cancer biomarker

## Abstract

**Background & Objective:**

Squamous cell carcinoma (SCC) is the second most common non-melanoma skin cancer that may be caused by *Human papillomavirus* (HPV), especially in immunosuppressed patients. However, the role of the mucosal types of HPV in SCC patients with normal immunity has not been extensively confirmed. The aim of this study was to investigate the association of some high-risk mucosal types of HPV with cutaneous SCC in an Iranian population.

**Methods:**

Sixty-five formalin-fixed, paraffin-embedded tissue specimens with a diagnosis of cutaneous SCC as the case group and sixty-five healthy skin specimens as the control group were included in our case-control study. Genomic DNA was extracted from tissue samples and then PCR was used for the detection of HPV genotypes by a commercial kit.

**Results:**

Our data revealed that 6 out of 65 SCC samples (9.2%) were infected by high-risk mucosal types of HPV whereas none of the 65 control samples were infected by the mentioned HPVs. Statistical analysis showed a significant association between these types of HPV infection and SCC risk in our studied population (*P*=0.028).

**Conclusion:**

These findings suggested that some high-risk mucosal types of HPV are significant risk factors for cutaneous SCC.

## Introduction

Squamous cell carcinoma (SCC) is the second most common skin malignancy after basal cell carcinoma (BCC), and its prevalence is increasing in many countries (1). SCC affects every part of the skin and mucosa with squamous epithelium, especially areas exposed to sunlight, such as the face, hands, and arms (2). Other risk factors of this disease are light skin, aging, chronic ulcers, male gender, actinic keratosis as a precursor of skin cancer, and a *Human papillomavirus* (HPV) infection (3).

HPV is a member of the *papillomaviridae* that has a circular double-stranded DNA with approximately 8kbp length (1), and consists of more than 100 different types of viruses that target squamous cells (4, 5). Oncoproteins E6 and E7 of this virus prevent apoptosis by binding to the P53 and pRb tumor suppressor genes in target cells, thus playing a pathogenic and progressive role in malignant changes (6). High-risk types of HPV cause cervical, anogenital, and oropharyngeal cancers, whereas cutaneous types are mainly related to benign and malignant skin lesions; some mucosal HPV types have been detected in skin malignancies (5). HPV18, 16, and 56, the common types of high risk mucosal HPV, have been frequently found in non-melanoma skin cancers (4, 7-9). Although the main cause of non-melanoma skin cancer is exposure to UV radiation, cutaneous HPV can also contribute to carcinogenesis with UV radiation (10). 

Studies in immunodeficient individuals have confirmed the role of HPV in skin SCC, but this effect has not yet been extensively studied in people with normal immunity (4). There is a strong association between HPV 5 and HPV 8, and the incidence of non-melanoma skin cancers in Epidermodysplasia verruciformis (EV), in which individuals are susceptible to multiple skin warts, and in which about 30-40% of infected individuals develop squamous cell carcinoma in areas of the skin exposed to the sun (11). In order to tackle the prevalence of SCC, the need for further research on its causes in an effort to find effective strategies for prevention and treatment, we investigated the association of high-risk HPVs with SCC in an Iranian population. 

## Materials and Methods

**Samples**

This case-control study was performed on the paraffin blocks of 65 patients with SCC as the case group and 65 healthy pilonidal sinus samples as the control group. Participants were referred to the pathology department of Shahid Beheshti Hospital, Kashan University of Medical Sciences (Kashan, Iran) during the years 2011-2017 and SCC diagnosis was confirmed by the pathologist. To control the main confounding variable, subjects with immunosuppressive diseases, a history of transplantation, and immunodeficiency were excluded from the study. Also, other possible confounding variables such as age, sex, and location of the lesion were matched between healthy and patient groups.

**DNA Extraction**

The most suitable paraffin blocks were selected, then 10 cross-sectional cuts of each block with a thickness of 3-5 μm were prepared under sterile conditions by microtome. After transferring the samples to microtubes and deparaffinization, DNA extraction steps were performed according to the DNA-Sorb-C kit’s (AmpliSens biotechnologies, Russia) instruction manual.

**Detection of HPV Genotypes**

After DNA extraction, 14 high-risk HPV genotypes were detected using an AmpliSens® HPV HCR genotype-titre-FRT PCR kit variant FRT-100 (AmpliSens®/Russia) within a DT-96 Real-Time PCR Cycler (DNA- Technology, Russia). The Beta-globin gene was used as an internal control to ensure proper DNA extraction. To carry out Real-time PCR, the entire volume of Taq F (60 μL) solution was initially mixed with 600 μL of PCR buffer B. Then 5μL of this mixture was added to 10 μL of four types of PCR mix F1 HPV (mix 16, 18, 31, Glob; mix 39, 45, 59, Glob; mix 33, 35, 56, 68; mix 51, 52, 58, 66). Of the four prepared solutions, 15 μL was added to each micro-tube containing DNA samples. Data analysis was performed using real-time PCR software by measuring the fluorescence signals obtained from 4 channels as follows: The DNA amplification signal for HPV types 58, 33, 39, 16 was identified in the FAM fluorescence channel. The DNA amplification signal for HPV types 52, 35, 45, 31 was detected in the JOE fluorescence channel. The DNA amplification signal for HPV types 66, 68, 59, 18 was identified in the ROX fluorescence channel. The DNA amplification signal for HPV types 51, 56 appeared in the Cy5 fluorescence channel. Finally, the internal control signal was detected by the Cy5 fluorescence channel in the microtubes containing PCR-mix F1 HPV 16, 18, 31/Glob and PCR-mix-F1 HPV 39, 45, 59/Glob.

**Statistical Analysis**

Data was collected and entered into SPSS version 19.0 (SPSS Inc., IBM Corp. Armonk, NY, USA). Independent t-test was used to analyze numerical data. Differences between the case and control groups regarding qualitative data, as well as genotype frequencies, were analyzed by χ2 test. P-values less than 0.05 were considered statistically significant.

## Results

Of all patients with SCC, 53 (81.5%) and 12 (18.5%) individuals were respectively male and female, which indicates a higher incidence of SCC in men. In addition, the most common site of SCC lesions was observed in the head, neck, face, and hands, which are areas exposed to sunlight (87%). Out of 65 patients with SCC in this study, 6 (2.9%) were HPV-positive, while none of the controls were positive (0.0%). Statistical analysis showed significant differences between case and control groups regarding HPV infection (*P*<0.05).

Of 14 high-risk studied HPV genotypes (16, 18, 31, 33, 35, 39, 45, 51, 52, 56, 58, 59, 66, 68), HPV 51 and 52 were observed in two SCC subjects but HPV 31 and 16 were observed in one SCC subject. Also, the most common HPV-positive sites of SCC lesions were the neck and face (Table 1). Of all patients with SCC who were also HPV-positive, four and two cases were respectively male and female (Table 1).

**Table 1:** HPV virus frequency and its genotypes in the SCC group based on the location of the lesion and gender

Lesion Region	Total No.	HPV positive		HPV negative	
		No (genotypes)	%	No.	%
Head, Neck & Face	50	5 (51, 52, 52, 31, 16)	10	45	90
Hand	7	0	0	7	100
Foot	4	1 (51)	25	3	75
Knee & Thigh	3	0	0	3	100
Chest	1	0	0	1	100
Gender					
Male	53	4 (31, 51, 52,52)	7.55	49	92.45
Female	12	2 (51, 16)	16.67	10	83.33

## Discussion

In this study, we investigated the presence of HPV high-risk types in patients with squamous cell carcinoma. Our study showed 9.23% of all patients with SCC were HPV-positive while none of the control group was HPV-positive. As seen, the incidence of HPV-infected cases in SCC patients was significantly higher than in healthy skin samples. According to previous studies, Shayanfar *et al.* (2013) found the DNA of HPV 18, 11, and 6 in 30% of malignant specimens of SCC while this level was reported to be 11% in controls that suggest an association between HPV infection and SCC risk (4). In a study on paraffin blocks of non-melanoma skin cancers and benign skin lesions, Shahmahmoudi* et al. *(2007) identified the DNA of HPV 18, 16, and 56 in 25.7% of malignant samples compared with 0.7% of benign ones (5). In a study on SCC biopsy specimens, Iftner* et al. *(2003) observed the DNA of HPV 16, 31, 35, and 51 in 59.7% of patients and 4.7% of controls (9). 

As mentioned, in some studies there was a significant association between HPV infection and SCC risk (4, 5). Also, the results of our study showed the possible role of HPV, especially types 16, 31, 51 and 52, in the development of SCC in Iranian patients. In some studies investigating the cutaneous HPV, there was a significant association between HPV infection and SCC risk (11-14) but some other studies reported no significant association (15-17). It seems that the inconsistent outcomes in different studies may be due to several factors such as the HPV genotypes (cutaneous or mucosal) and genotypes, virus identification method (serology or tissue study), sample type (paraffin blocks or fresh tissue), epidemiological factors, and the prevalence of HPV in different geographical regions. The controversial outcomes of HPV association with SCC require further studies to arrive at more comprehensive results. 

There are some limitations in our study which should be noted. At first, we did not evaluate the cutaneous HPV types in our studied population. Also, we did not assess the high-risk HPV in benign skin lesions. In addition, we only employed the real-time PCR for HPV typing. However, proving the association between HPV and cutaneous SCC requires more experimental studies to determine the transcriptional activity of HPV in squamous cell carcinoma. Serological studies, HPV protein marker detection, and PCR being used simultaneously on SCC specimens, as well as studies on more patients in different geographical regions, can provide a better perspective of HPV’s role in SCC.
